# Behavior Characteristics and Thermal Energy Absorption Mechanism of Physical Blowing Agents in Polyurethane Foaming Process

**DOI:** 10.3390/polym15102285

**Published:** 2023-05-12

**Authors:** Haozhen Wang, Yingshu Liu, Lin Lin

**Affiliations:** School of Energy and Environmental Engineering, University of Science and Technology Beijing, Beijing 100083, China

**Keywords:** polyurethane, physical blowing agent, efficiency, dissolution, loss rate, heat absorption

## Abstract

Polyurethane rigid foam is a widely used insulation material, and the behavior characteristics and heat absorption performance of the blowing agent used in the foaming process are key factors that affect the molding performance of this material. In this work, the behavior characteristics and heat absorption of the polyurethane physical blowing agent in the foaming process were studied; this is something which has not been comprehensively studied before. This study investigated the behavior characteristics of polyurethane physical blowing agents in the same formulation system, including the efficiency, dissolution, and loss rates of the physical blowing agents during the polyurethane foaming process. The research findings indicate that both the physical blowing agent mass efficiency rate and mass dissolution rate are influenced by the vaporization and condensation process of physical blowing agent. For the same type of physical blowing agent, the amount of heat absorbed per unit mass decreases gradually as the quantity of physical blowing agent increases. The relationship between the two shows a pattern of initial rapid decrease followed by a slower decrease. Under the same physical blowing agent content, the higher the heat absorbed per unit mass of physical blowing agent, the lower the internal temperature of the foam when the foam stops expanding. The heat absorbed per unit mass of the physical blowing agents is a key factor affecting the internal temperature of the foam when it stops expanding. From the perspective of heat control of the polyurethane reaction system, the effects of physical blowing agents on the foam quality were ranked in order from good to poor as follows: HFC-245fa, HFC-365mfc, HFCO-1233zd(E), HFO-1336mzzZ, and HCFC-141b.

## 1. Introduction

Polyurethane rigid foam (PURF) finds extensive use in white goods, cold storage, and construction due to its excellent thermal insulation performance [1,2,3]. The production process of PURF requires the use of blowing agents (BA), the most important of which are physical blowing agents (PBAs), which produce gas to achieve a foaming effect by physical phase change [4]. PURF needs to undergo a complex process from liquid to solid during the foaming process. In this process, the change of blowing agent is particularly important. PBAs exist in two typical states in the polyurethane foaming process, namely the vaporization state and the dissolution state. In this process, there will also be a certain amount of mass lost from the PBAs.

During the foaming process, most PBAs promote foam expansion when in a gaseous state. The efficiency of PBAs is defined as the proportion of PBA molecules that contribute to foam expansion out of the total PBA molecules. Some PBAs in the gaseous state escape into the air during foaming, and the proportion of these escaped PBA molecules out of the total PBA molecules is referred to as the loss rate of PBAs. After foaming is complete, dissolved PBAs remain in the polyurethane foam matrix, and the proportion of these dissolved PBA molecules out of the total PBA molecules is known as the dissolution rate of PBA. The efficiency of PBAs is a crucial criterion for determining the suitability of PBA in industrial applications, as it significantly affects the cost, foaming efficiency, and process performance. The loss rate of PBAs is closely related to the environmental friendliness of the foaming process and affects the cost of the final product. Due to the high-ozone-depletion potential (ODP) [5] of second-generation PBAs and the high-global-warming potential (GWP) [6] of third-generation PBAs, each can have a certain impact on the environment. The dissolution rate of PBAs is closely related to the quality of the foam product, as the PBAs remaining in the resin matrix often act as plasticizers, adversely affecting the thermal conductivity, compressive strength, dimensional stability, fire resistance, and adhesion to the substrate of the foam.

In previous studies, we have developed a model to measure the PBA loss rate and efficiency in PURF using systematic experiments [7,8]. According to the law of mass conservation, we can further calculate the PBA dissolution rate in foam. Due to the endothermic phase change characteristic of PBAs, their mass transfer and endothermic processes have a great influence on the reactivity, moldability, and various physical properties of polyurethane foam after foam formation.

Previous studies on the PURF foaming process have mostly focused on bubble formation, growth, and modeling based on reaction kinetics. Few scholars have focused on the behavior of PBAs and their impact on the system’s heat absorption. Nucleation and growth were the focus of previous studies, among which the former provided the initial conditions for the latter. However, the actual quantity of PBAs used in this process was not considered by previous researchers [9,10,11]. In terms of reaction kinetics modeling, Rojas et al. [12] considered the foaming kinetics as controlled by the rate of heat generation. The basic theoretical ideas of Rojas’ study were cited and improved by many subsequent researchers. Baser et al. [13,14] refined Rojas’ model by considering the dilution effect of the blowing agent on the reaction mixture, while Tesser et al. [15] further optimized the model to include heat transfer and modified the vapor–liquid equilibrium description of the blowing agent and polymer phases using an extended Flory–Huggins equation. Zhao et al. [16] first reported that the method of solving ordinary differential equations using a MATLAB program was used to establish a theoretical model to simulate the foaming process of PURF. Consequently, Al-Moameri et al. [17] used a MATLAB simulation code to simulate the kinetic and physical properties of the polyurethane foaming process. Harikrishnan et al. [18] studied a kinetic simulation of polyurethane foam reaction and film thinning. Suppes et al. [19] studied a simulation of urethane reaction processes and foaming processes for an analytical method. Moameri, Harith Al et al. [20] roughly studied the residual amount (dissolution rate) of some PBAs in the resin phase by means of baking loss and compared the simulation values with the experimental data. However, high-temperature baking can only cause partial loss of PBAs, and the decomposition loss of polyurethane foam at high temperatures will also bring significant errors to the experiment. Shen et al. [21] quantitatively modeled the foam density of the physical process of polyurethane box foaming. Gandhi et al. [22] developed a model forecast the density distribution of polyurethane foams blown by water as a chemical blowing agent. Raimbault et al. [23] developed an analytical model for polyurethane foams based on FOAMAT^®^ experiments, which was verified using finite element software.

These studies have gradually deepened our understanding of the polyurethane reaction process and laid a foundation for further predicting the reaction. However, as of yet, there has been no systematic study of the behavior characteristics of PBAs and the impact of PBA systems on the heat of the polyurethane reaction. The deficiencies in this research field affect the accuracy and applicability of modeling and calculations to a certain extent. This study tested for the first time the efficiency, dissolution, and loss rates of second-, third-, and fourth-generation PBAs (HCFC-141b, HFC-245fa, HFC-365mfc, HFCO-1233zd(E), and HFO-1336mzzZ [24]) in the same formulation system. The heat absorption rates of the five PBAs per-unit-mass were systematically calculated in the reaction system.

In the complex chemical reaction and physical change process of polyurethane foaming, the heat absorption of the PBAs is a key factor that controls the process. This can be achieved by changing the amount and type of PBA added. By studying the heat absorption of different PBA systems, this study provides reference information for adjusting polyurethane formulations. The results can guide the optimization and control of the reaction process and are of great significance for improving foamability and controlling the total heat of the reaction.

## 2. Modeling Methods

### 2.1. Measurement of PBA Efficiency, Dissolution, and Loss Rate during Foaming Process

In previous research, we developed a model for measuring the loss rate and efficiency of PBAs in PURF foaming process through numerous experiments and systematic design. We used the method of adding water to replace the difference to accurately measure the volume of polyurethane foam, which can solve the problem of irregular growth shape during foaming process [8].

Based on accurate measurement of foam volume, we calculated the buoyancy of humid air during the foam expansion process. After further experimental elimination of the contribution of small molecule substance loss in the foam, we were able to calculate the actual mass loss of PBAs [8]. By measuring the foaming pressure and temperature when the foam volume stops expanding, we established a basic method for calculating the effective mass of PBA. After further experimental removal of the contribution of CO_2_ generated by water reacting with isocyanate and calculation correction of the compressibility coefficient of various PBA gases, we were able to calculate the effective mass of PBA in the foaming process [7]. After calculating the loss mass and effective mass of PBA in the same polyurethane foam system, we can calculate the dissolution mass of PBA in the polyurethane system according to the law of mass conservation. Based on this, we can further calculate the mass loss rate, mass efficiency rate, and mass dissolution rate of PBA in the same polyurethane foam system.

In this study, we adopted the methodology directly from our previous work (Wang, H.Z. et al., Changes and Trends—Efficiency of Physical Blowing Agents in Polyurethane Foam Materials [7] and Wang, H.Z. et al., Eco-Friendly Physical Blowing Agent Mass Loss of Bio-Based Polyurethane Rigid Foam Materials [8]), which was thoroughly described. As no modifications were made to the methodology, we refer the reader to those publications for a comprehensive understanding of the procedure.

### 2.2. Measurement of Heat Absorption of PBA during the Foaming Process

**Figure 1:**
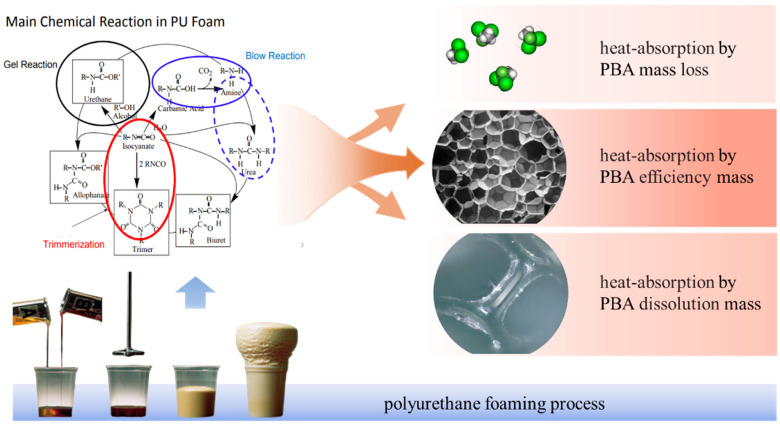
The distribution of heat absorbed by PBA.

During the foaming process, the heat absorbed by PBA can be divided into three parts: (a) the heat carried away by the escaping PBA from the foam surface (heat absorption by PBA mass loss), which includes both the latent and sensible heat of PBA; (b) the heat absorbed by the PBA that causes the expansion of the foam volume (heat absorption by PBA efficiency mass), which also includes the latent and sensible heat of PBA; (c) the heat absorbed by the PBA dissolved in the foam structure (heat absorption by PBA dissolution mass), which is mainly the sensible heat absorbed by PBA during the foam formation process. If $Q_{PBA}$ (J) represents the total heat absorption by PBA during the foaming process, it can be expressed in the form of Equation (1).

(1) $$Q_{PBA}=Q_{l}{+Q}_{e}+Q_{d}$$

where $Q_{l}$ (J) represents the heat absorption by PBA mass loss; $Q_{e}$ (J) represents the heat absorption by PBA efficiency mass; $Q_{d}$ (J) represents the heat absorption by PBA dissolution mass. The distribution of heat absorbed by PBA is shown in Figure 1.

#### 2.2.1. Heat Absorption by PBA Mass Loss

Heat absorption by PBA mass loss is represented by Equation (2), where γ (J·g^−1^) is the latent heat of vaporization of PBA at the temperature $T_{0}$ (K), which is the initial foaming temperature. $m_{l}$ (g) is the PBA mass loss. $C_{g0}$ (J·g^−1^·K^−1^) is the specific heat capacity of PBA gas at the temperature of $T_{0}$ (K), and $C_{g1}$ (J·g^−1^·K^−1^) is the specific heat capacity of PBA gas at the temperature of $T_{1}$ (K). $T_{1}$ (K) is the temperature of PBA at the surface escape.

(2) $$Q_{l}=\gamma m_{l}+m_{l}({C_{g1}T}_{1}-C_{g0}T_{0})$$

Since the process of PBAs escaping through the foam surface is continuous and its temperature is not fixed, an accurate value of $Q_{l}$ (J) cannot be measured directly. However, the enthalpy of PBA vaporization is much greater than its specific heat capacity at constant pressure, and the surface temperature of the polyurethane in contact with air is not high when PBAs gasify and escape internally. Furthermore, the mass loss of PBAs during foaming is typically less than 8% of the total PBAs used. Therefore, the specific heat term $m_{l}({C_{g1}T}_{1}-C_{g0}T_{0})$ carried away by PBAs in $Q_{l}$ (J) can be ignored during the calculation process. This simplification has a negligible effect on the total heat absorbed by PBA during the foaming process, $Q_{PBA}$ (J).

#### 2.2.2. Heat Absorption by PBA Efficiency Mass

Heat absorption by PBA efficiency mass is represented by Equation (3), where $m_{e}$ (g) is the PBA efficiency mass. $C_{gF}$ (J·g^−1^·K^−1^) is the specific heat capacity of PBA gas at the temperature of $T_{F}$ (K). $T_{F}$ (K) is the temperature inside the foam when it stops expanding.

(3) $$Q_{e}=\gamma m_{e}+m_{e}({C_{gF}T}_{F}-C_{g0}T_{0})$$

The viscosity change trend in the polyurethane foaming process has the following characteristics: the viscosity is very low in the initial stage of the reaction and increases very slowly, and then rapidly increases as it approaches gelation, and the foam solidifies into a solid [16]. During the polyurethane foaming process, the internal pressure of the foam at the cessation of growth is typically below 1.11 × 10^5^ Pa [7]. In contrast, the critical temperatures and pressures of the PBAs are shown in Table 1 and are much higher than the pressures experienced during foaming. Therefore, the pressure within the foam during the process is significantly lower than the critical values of PBAs.

It has been found that the compression factors Z for PBAs transforming into the gas state within the foam are close to 1 (ranging from 0.980 to 0.995) [7], indicating that the gas can be treated as an ideal gas. Therefore, it is appropriate to assume that the specific heat capacity of PBAs in the gas state, $C_{g}$ (J·g^−1^·K^−1^), can be approximated using the specific heat capacity at constant pressure in their ideal gas state.

#### 2.2.3. Heat Absorption by PBA Dissolution Mass

Heat absorption by PBA dissolution mass is represented by Equation (4), where $m_{d}$ (g) is the PBA dissolution mass, $C_{L0}$ (J·g^−1^·K^−1^) is the liquid specific heat capacity of the PBA at the temperature of $T_{0}$ (K), and $C_{LF}$ (J·g^−1^·K^−1^) is the liquid specific heat capacity of the PBA at the temperature of $T_{F}$ (K).

(4) $$Q_{d}=m_{d}({C_{LF}T}_{F}-C_{L0}T_{0})$$

In general, changes in pressure have little effect on the heat capacity of solids, while for liquids, significant effects on heat capacity occur only near the critical point [25]. Since the actual pressure generated by the foam during the polyurethane foaming process is much lower than the critical pressure of the PBAs, the liquid heat capacity of the PBAs, $C_{L}$ (J·g^−1^·K^−1^), can be approximated using their constant pressure specific heat capacity value at the corresponding temperature, and its influence on the calculation results can be ignored.

#### 2.2.4. Specific Heat Capacity at Constant Pressure of PBA in the Ideal Gas State and in the Liquid State

The liquid isobaric specific heat capacity of PBA is calculated by the Sternling–Brown equation [26], as shown in Equation (5). The ideal gas state isobaric specific heat capacity of PBA is calculated by Equation (6) [27].

(5) $$\frac{C_{p}-C_{p}^{\prime }}{R}=(0.5+2.2\omega )[3.67+11.64(1-T_{r}{)}^{4}+0.634(1-T_{r}{)}^{-1}]$$

(6) $$\frac{C_{p}^{\prime }}{R}=A+BT+CT^{2}+DT^{3}+ET^{4}$$

where $C_{p}^{\prime }$ (J·g^−1^·K^−1^) is the specific heat capacity at constant pressure for PBAs in their ideal gas state, $C_{p}$ (J·g^−1^·K^−1^) is the specific heat capacity at constant pressure for PBAs in the liquid state. $R_{}$ = 8.314 J·mol^−1^·K^−1^ is the gas constant, ω is the eccentricity factor, and $T_{r}$ is the contrast temperature.

A, B, C, D, and E are corresponding proportion coefficients. Currently, only the relevant data for CO_2_ and HCFC-141b can be found in publicly available databases. The relevant proportion coefficients for the other four PBAs need to be fitted based on experimental data provided by the manufacturer, and the specific values will be explained in the Analysis and Discussion section.

#### 2.2.5. The Specific Heat of Vaporization of PBA at Different Temperatures

The specific heat of vaporization of PBA at its boiling point is relatively easy to obtain, but the specific heat of vaporization at other temperatures needs to be further calculated. The Watson equation [28,29] is a useful tool for calculating the heat of phase change at other temperatures, given the heat of phase change at a known temperature. This equation takes into account the different molecular internal energy of liquid at different temperatures, which affects the specific heat of vaporization during phase change. When the difference between the temperature and the critical temperature is greater than 10 K, the Watson equation has been found to have an error of only 1.8%. This makes it a valuable tool for approximating the specific heat of vaporization at other temperatures and it is used in our calculation process. The Watson formula is shown in Equation (7).

(7) $$\frac{(\Delta h_{v}{)}_{2}}{(\Delta h_{v}{)}_{1}}=(\frac{1-T_{r2}}{1-T_{r1}}{)}^{0.38}$$

where $(\Delta h_{v}{)}_{1}$ (J·g^−1^) and $(\Delta h_{v}{)}_{2}$ (J·g^−1^) represent the enthalpy changes in states 1 and 2, respectively, corresponding to the latent heat of phase change. $T_{r1}$ and $T_{r2}$ are the temperatures in states 1 and 2, respectively, which are being compared.

#### 2.2.6. Calculation of the Heat Absorbed during the Foaming Process of Unit Mass of PBA

According to the above analysis, the total heat absorbed by the PBA during the foaming process after the corresponding substitution and merger of Equation (1) can be expressed by Equation (8):

(8) $$Q_{PBA}=\gamma (m_{l}+m_{e})+m_{e}({C_{pF}^{\prime }T}_{F}-C_{p0}^{\prime }T_{0})+m_{d}({C_{pF}T}_{F}-C_{p0}T_{0})$$

where $C_{p0}^{\prime }$ (J·g^−1^·K^−1^) is the ideal gas specific heat capacity of the PBA at temperature $T_{0}$ (K), $C_{pF}^{\prime }$ (J·g^−1^·K^−1^) is the ideal gas specific heat capacity of the PBA at temperature $T_{F}$ (K), $C_{p0}$ (J·g^−1^·K^−1^) is the liquid specific heat capacity of the PBA at temperature $T_{0}$ (K), and $C_{pF}$ (J·g^−1^·K^−1^) is the liquid specific heat capacity of the PBA at temperature $T_{F}$ (K).

The heat absorbed per unit mass of PBA, denoted as $Q_{m}$ (J·g^−1^), is defined by Equation (9), where $m_{PBA}$ is the initial PBA mass. The initial mass ratio of PBA to other mixture materials in PURF is w (wt%), and w is defined by Equation (10). Where $m_{c}$ (g) is the material total mass of the foaming start without PBA.

(9) $$Q_{m}=\frac{Q_{PBA}}{m_{PBA}}$$

(10) $$w=\frac{m_{PBA}}{m_{c}}\times 100\%$$

## 3. Experimental

### 3.1. Raw Materials and Experimental Apparatus

The following chemicals and equipment were used in this study: PM-200, an oligomeric isocyanate of 4-4′ diphenyl methane diisocyanate with an average functionality of 2.7, and NCO% of 31%, obtained from Wanhua Chemical Group Co., Ltd.; R4110A, a polyol with a hydroxyl number of 440 mg KOH/g, functionality of 4.3, and average molecular weight of 550, obtained from Jining Baichuan Chemical Co., Ltd.; DMCHA, PC41, and DBTDL, catalysts, obtained from Beijing Intech Co., Ltd.; BL-8950, a silicone-type surfactant, obtained from Shanghai Menhover Chemical Company Limited; TCPP, a flame retardant for PURF, obtained from Jiangsu Yoke Technology Co., Ltd.; and blowing agents HCFC-141b, HFC-245fa, HFC-365mfc, HFCO-1233zd(E), and HFO-1336mzzZ, obtained from Changshu San’aifu Fluorine Chemical Co., Ltd., Honeywell (China), Co., Ltd., Solvay Co., Ltd., Honeywell (China), Co., Ltd., and Chemours Chemistry (Shanghai, China) Co., Ltd., respectively.

The equipment used included a high-speed frequency conversion dispersing machine (JFS1100-ST, from Hunan Lichen Instrument Technology Co., Ltd.), an electronic balance (JJ2000B, from Hangzhou Shuangjie Electronic Computer Co., Ltd.), a miniature plane capsule pressure sensor (JHHM-H3, from Bengbu Jinnuo Sensor Co., Ltd.), a pressure sensor display instrument (JH-808, from Bengbu Jinnuo Sensor Co., Ltd.), a thermocouple temperature measuring wire (TT-T-30, from Xinghua Suma Electric Instrument Co., Ltd.), a high-precision portable thermocouple thermometer (YET-640, from Xinghua Suma Electric Instrument Co., Ltd.), a barometer (DYM-3, from Shanghai Instrument Technology Co., Ltd., Shanghai, China), and a hygrograph (GJWS-T1, from Shanghai Instrument Technology Co., Ltd., Shanghai, China).

### 3.2. Experimental Procedure

The foaming recipes used in the experiment are shown in Table 2, in which all “PBA*” dosages were tested using HCFC-141b, HFC-245fa, HFC-365mfc, HFCO-1233zd(E), and HFO-1336mzzZ.

At room temperature, mix the components except for PM200 according to the proportions given in Table 2 and use them as A-side backup. During foaming, an appropriate amount of A-side and corresponding proportion of B-side were placed into the same beaker. The mixture was stirred at 2500 rpm for 10 s, and then the contents were quickly poured into a plastic beaker. During and after foaming, record the value required and measure the mass of the water added. The specific data that need to be measured and recorded include the total material mass at the start of foaming, total material mass after foaming, the mass of water added to the empty beaker at the scale mark, and the mass of water added to the mark on the beaker after foaming, among others. Repeat the previous mixed foaming operation. A pressure sensor and a thermocouple are used to measure the pressure and temperature when the foam stops expanding [7,8].

## 4. Results and Discussion

### 4.1. The Mass Efficiency Rate, Mass Dissolution Rate and Mass Loss Rate of PBAs

**Figure 2:**
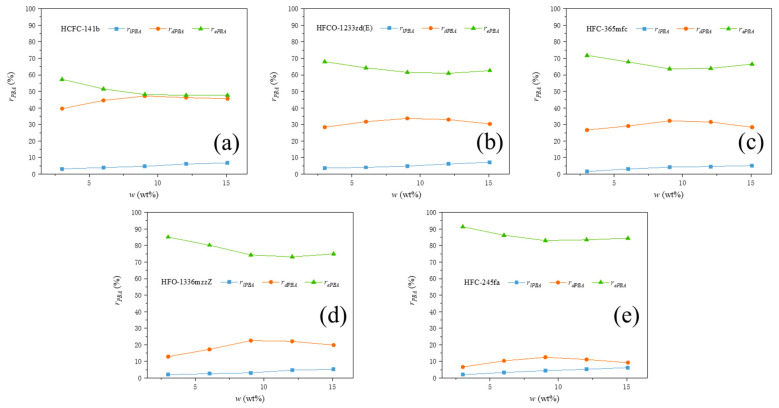
The ratio of each PBA’s three states to the total mass of PBAs used. In which the PBA systems were (**a**) HCFC-141b, (**b**) HFCO-1233zd(E), (**c**) HFC-365mfc, (**d**) HFO-1336mzzZ, and (**e**) HFC-245fa.

**Figure 3:**
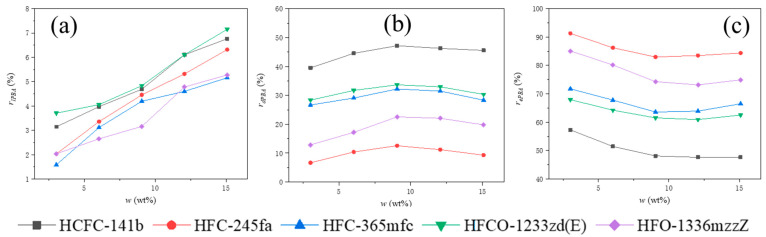
Comparison chart of (**a**) PBA loss rate $r_{lPBA}$ (**b**) PBA dissolution rate $r_{dPBA}$, and (**c**) PBA efficiency rate $r_{ePBA}$.

Using the method described previously, relevant experiments were conducted on the recipes listed in Table 2 to obtain the mass efficiency rate, mass dissolution rate, and mass loss rate of the five PBAs used at different dosages. The ratio of each PBA’s three states to the total mass of PBAs used, denoted as $r_{PBA}$ (%), is shown in Figure 2. The performance of the five PBAs was compared in terms of PBA loss rate ($r_{lPBA}$), PBA efficiency rate ($r_{ePBA}$), and PBA dissolution rate ($r_{dPBA}$), as shown in Figure 3.

It can be seen that the efficiency, dissolution, and loss rate of different PBAs under the same experimental conditions vary greatly in Figure 2 and Figure 3. The smallest difference is observed in the loss rate, and the proportion of the loss rate $r_{lPBA}$ of all five PBAs relative to the total amount of PBA used is relatively low, ranging from 1% to 7%. Different PBAs show significant differences in both efficiency and dissolution. The overall average efficiency rate $r_{ePBA}$ ranges from around 50% to around 85%. The average efficiency rates $r_{ePBA}$ of the five PBAs in descending order are: HFC-245fa, HFO-1336mzzZ, HFC-365mfc, HFCO-1233zd(E), and HCFC-141b. The overall average dissolution rate $r_{dPBA}$ ranges from around 10% to 45%. The average dissolution rates $r_{dPBA}$ of the five PBAs in descending order are: HCFC-141b, HFCO-1233zd(E), HFC-365mfc, HFO-1336mzzZ, and HFC-245fa. Since the proportion of the PBA loss rate is small, there is a clear correlation between PBA efficiency and dissolution rate, both of which are related to the equilibrium state of PBA vaporization and condensation. When the foam growth stops, the two reach a dynamic equilibrium. At this time, the number of PBA molecules escaping from the foam skeleton by vaporization is the same as the number of PBA molecules that have already undergone vaporization and condensation to enter the foam skeleton from the pores.

From Figure 2 and Figure 3, it can be seen that the loss rates $r_{lPBA}$ of all experimental groups of PBAs show a continuous increasing trend as w (the initial mass ratio of PBA to other blending materials in PURF) increases. The dissolution rate $r_{dPBA}$ of all experimental groups of PBAs initially shows an increasing trend and then gradually decreases as w increases. The efficiency rates $r_{ePBA}$ of all experimental groups of PBAs show a decreasing trend at first and then gradually stabilize or slightly increase as w increases. These trends indicate that different PBAs will have the same influencing factors as their addition amounts change in the foaming material. As w increases, the number of PBA molecules in the foaming material gradually increases, and the influence of PBA molecules and their interactions with other components in the foaming material during volatilization and vaporization processes increases.

Regarding the process of PBA volatilization loss, it refers to the diffusion process of PBA molecules penetrating through the foam surface and evaporating into the air. During this process, the foam surface is always in contact with the air, so its contact surface temperature with the air is significantly lower than the internal temperature of the foam and is close to the ambient air temperature. Increasing the PBA content has a significant impact on the heat and temperature inside the foam, but little effect on the foam surface temperature. The influence of PBAs in the foaming material by the interaction of the same PBA molecules and other components in the foaming material has always been the dominant factor, so all experimental groups’ PBA loss rate $r_{lPBA}$ show a positive correlation with the increase of w.

**Figure 4:**
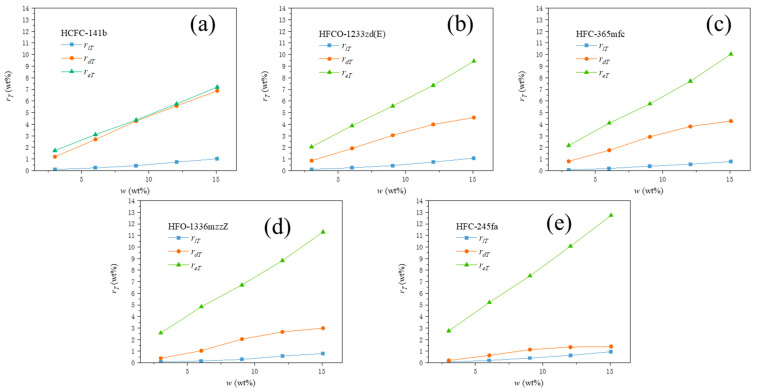
The ratios of the three states of each PBA relative to the total mass of material used, $r_{T}$ (wt%). In which the PBA systems were (**a**) HCFC-141b, (**b**) HFCO-1233zd(E), (**c**) HFC-365mfc, (**d**) HFO-1336mzzZ, and (**e**) HFC-245fa.

For the vaporization and dissolution equilibrium process of PBA, this process occurs inside the foam body. Wang W L et al. have demonstrated that during the foaming process, the temperature inside the foam remains uniformly rising, and the foam temperature near the boundary gradually approaches the external temperature (room temperature). When the PBA content is low, the amount of heat absorbed by PBAs in the foaming system is low, and the foaming system heats up faster. The internal temperature of the foam corresponding to the cessation of expansion is higher when the foam stops expanding. For PBAs, it reaches the corresponding vaporization conditions earlier and is more easily sustained. This results in more effective PBA molecules to cause foam expansion. Its efficiency rate, $r_{ePBA}$, is higher, while the dissolution rate, $r_{dPBA}$, is lower. As the PBA content increases, the amount of heat absorbed by PBAs in the foaming system increases, and the rate of temperature rise in the foaming system slows down. The internal temperature of the foam corresponding to the cessation of expansion, $T_{F}$, decreases. All of these conditions have an adverse effect on the sustained vaporization of PBA. Its efficiency rate, $r_{ePBA}$, gradually decreases, while the dissolution rate, $r_{dPBA}$, gradually increases. As the PBA content continues to increase, the interaction between PBA molecules in the system and its interaction with other component molecules in the foaming material gradually strengthens. Under the same conditions, the absolute number of PBA molecules that produce effective vaporization increases accordingly. This condition has a favorable effect on the vaporization of PBA. Under the combined effect of the internal foam temperature, $T_{F}$, and the interaction of PBA molecules, the efficiency rate, $r_{ePBA}$, shows a trend of decreasing first and then gradually stabilizing or slightly increasing with increasing w, while the dissolution rate, $r_{dPBA}$, shows a trend of increasing first and then gradually decreasing with increasing w. The significant decrease in the dissolution rate, $r_{dPBA}$, in the later stage is also related to the continuous increase in the PBA loss rate, $r_{lPBA}$, with increasing w. From the results of the two factors’ effects, it can be seen that when w is less than 9.05 wt%, the influence of system temperature is dominant, while when w is greater than 9.05 wt%, the interaction of PBA molecules in the system and its interaction with other component molecules in the foaming material is dominant. Figure 4 illustrates the ratios of the three states of each PBA relative to the total mass of material used in the foaming process, denoted as $r_{T}$ (wt%).

From Figure 4, it can be seen that the ratio of the three states of each PBA relative to the total amount of material used, $r_{T}$ (wt%), shows a clear positive correlation with w. This indicates that the concentration of PBA is the dominant controlling factor for the absolute amount of each state of PBA in the foaming process. The different changes in the ratio of the three states of different PBAs relative to the total amount of material used, $r_{T}$ (wt%), with w, indicate that their own properties will lead to significant differences in $r_{lT}$ (wt%), $r_{dT}$ (wt%), and $r_{eT}$ (wt%).

### 4.2. The Heat Absorption of PBA during the Foaming Process

#### 4.2.1. The Fitting of the Proportion Coefficients A, B, C, D and E of PBAs

**Figure 5:**
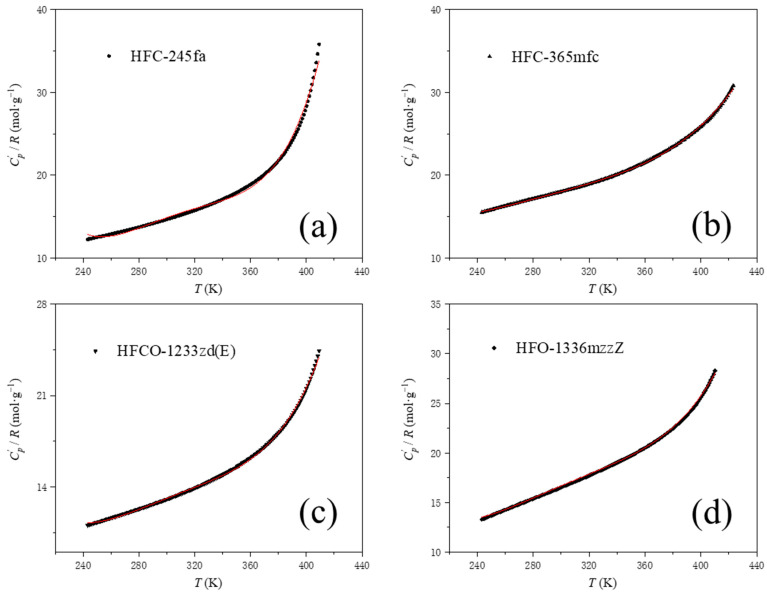
Fitting plot of the change curve of the specific heat capacity of the ideal gas with temperature of PBAs. (**a**) HFC-245fa data and fitted curve, (**b**) HFC-365mfc data and fitted curve, (**c**) HFCO-1233zd(E) data and fitted curve, and (**d**) HFO-1336mzzZ data and fitted curve. The solid red line in the figure is the fitted curve.

Based on the ideal gas constant pressure specific heat values of PBAs at different temperatures provided by manufacturers of HFCO-1233zd(E), HFC-365mfc, HFO-1336mzzZ, and HFC-245fa, the value of $\frac{C_{p}^{\prime }}{R}$ can be calculated. The curve of $\frac{C_{p}^{\prime }}{R}$ as a function of temperature can be fitted using a fourth-order polynomial to obtain the values of the five proportion coefficients A, B, C, D and E in Equation (6). The data provided by the PBAs’ manufacturers and the fitting results are shown in Figure 5.

The values of the five scale coefficients A, B, C, D and E for each of the fitted PBAs are shown in Table 3:

#### 4.2.2. The Specific Heat of Vaporization of PBA at Different Temperatures

According to the known specific heat of vaporization at the boiling point of PBAs (provided by the manufacturers), the specific heat of vaporization values of HCFC-141b, HFC-245fa, HFC-365mfc, HFCO-1233zd(E) and HFO-1336mzzZ at 293.15 K (the starting temperature of foaming) can be calculated using the Watson formula, as shown in Table 4.

#### 4.2.3. The Heat Absorbed during the Foaming Process of Unit Mass of PBA

The $T_{F}$ (K) of PBAs at different concentrations is shown in Table 5.

The ideal gas constant pressure specific heat and liquid constant pressure specific heat of PBAs at different concentrations can be calculated based on the corresponding $T_{F}$ (K) values of PBAs at different concentrations. Table 6 and Table 7 show the calculated results.

Table 6 and Table 7 demonstrate that the specific heat capacity at constant pressure of PBAs in both the ideal gas and liquid state exhibits a similar trend, which is a decrease with an increase in w (wt%). This is due to the relationship between $T_{F}$ (K) and w (wt%), where the specific heat capacity at constant pressure in both the ideal gas and liquid state decrease with decreasing temperature. The values of $C_{p}^{\prime }$ (J·g^−1^·K^−1^) and $C_{p}$ (J·g^−1^·K^−1^) for different PBAs vary greatly, mainly influenced by the relative molecular mass, boiling point, and critical parameters of the PBA itself.

**Figure 6:**
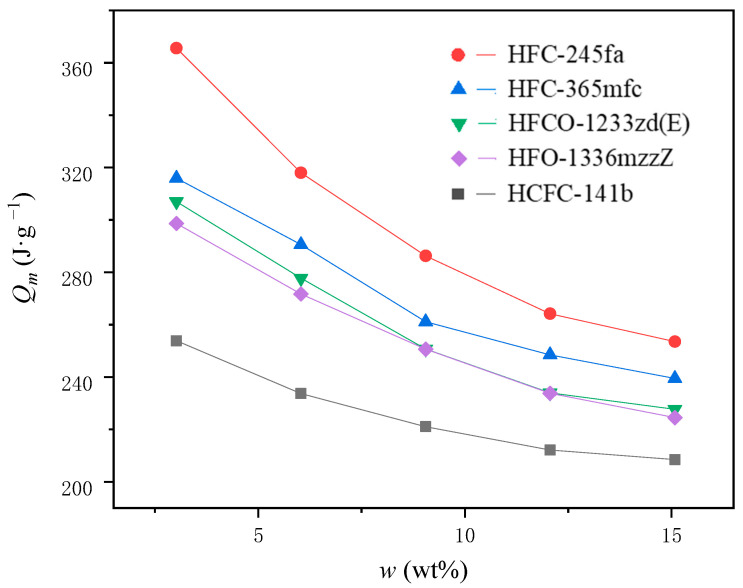
The heat absorbed per unit mass of PBAs at different concentrations.

By plugging in the aforementioned parameters into Equations (8) and (9), the heat absorbed by PBA per unit mass $Q_{m}$ (J·g^−1^) at different concentrations can be calculated, as depicted in Figure 6.

As shown in Figure 3, the amount of heat absorbed per unit mass of PBA, $Q_{m}$ (J·g^−1^), decreases gradually as the amount of PBA increases for the same type of PBA. Overall, the relationship between $Q_{m}$ (J·g^−1^) of all PBAs and w (wt%), shows a pattern of rapid decrease followed by a slower decrease. The reasons for this phenomenon can be attributed to the following three factors: (a) When PBA is added in smaller amount, the foaming temperature of the system is higher and the efficiency of PBA relative to the total mass of PBA used is higher [7]. Since the latent heat of PBA vaporization is much higher than its sensible heat in gas and liquid states, the increase in PBA efficiency results in higher heat absorption per unit mass of PBA. (b) The specific heat capacity of PBA in gas and liquid states increases with temperature. Therefore, in the same temperature difference range, the PBA with a higher temperature contains more heat energy per unit mass, resulting in higher heat absorption per unit mass of PBA. (c) When PBA is added in smaller amounts, it has less effect on the polyurethane reaction. The accumulation reaction of polyurethane foam systems releases heat faster and further accelerates the reaction, solidifying in less time and stopping expanding. Less heat is lost on the upper surface of the foam during the experiment, so the heat absorbed per unit mass of PBA increases.

For different PBAs, the thermal value $Q_{m}$ (J·g^−1^) absorbed by PBA per unit mass under the same concentration conditions was significantly different. It can be seen that there is a significant correlation between the heat absorbed by different PBA systems $Q_{m}$ (J·g^−1^) and $T_{F}$ (K). Under the same PBA content, the higher the heat absorbed $Q_{m}$ (J·g^−1^) per unit mass of PBA, the lower the internal temperature $T_{F}$ (K) of the foam when the foam stops expanding. The higher the amount of PBA added, the higher the heat absorbed by vaporization in the system and the sensible calorific value of the gaseous and liquid states absorbed by itself. In turn, the more heat is absorbed from the foaming system, the lower the internal temperature $T_{F}$ (K) of the foam when it stops expanding. The latent heat value of vaporization of different PBAs γ (J·g^−1^), the specific heat capacity of liquid isobaric $C_{L}$ (J·g^−1^·K^−1^) and gas isobaric specific heat capacity $C_{g}$ (J·g^−1^·K^−1^) are significantly different. The mass efficiency, dissolution, and loss rate of PBA in the actual foaming process also have obvious differences. Therefore, the calorific value absorbed by PBA per unit mass $Q_{m}$ (J·g^−1^) determined by the above factors also showed significant differences with PBA types. The heat absorbed by PBA per unit mass $Q_{m}$ (J·g^−1^) is a key factor affecting the internal temperature $T_{F}$ (K) of the foam when it stops expanding. $T_{F}$ (K) can be thought of as a measure of the final equilibrium state of the foam reaction exothermic and PBA endothermic.

The calorific absorption value per unit mass of different PBAs at different concentrations has an important influence on the heat production control and foaming process in the polyurethane foaming process. This problem directly affects the rate of foaming reaction and the maximum temperature during foam molding and has a great impact on whether there is heartburn during foaming and the physical properties of the molded foam, such as mechanical strength and thermal conductivity. From the perspective of heat control of polyurethane reaction system, HFC-245fa has the best effect, HCFC-141b has the worst effect, and HFCO-1233zd(E), HFO-1336mzzZ, and HFC-365mfc have similar effects, among which HFC-365mfc is slightly better than the other two.

## 5. Conclusions

This study tested for the first time the efficiency, dissolution, and loss rates of second, third, and fourth generation blowing agents HCFC-141b, HFC-245fa, HFC-365mfc, HFCO-1233zd(E), and HFO-1336mzzZ in the same formulation system. The correlation coefficients of the formulas for calculating the specific heat capacity of ideal gases at different temperatures of HFCO-1233zd€, HFC-365mfc, HFO-1336mzzZ, and HFC-245fa were also disclosed for the first time. On this basis the heat absorption rates of the five PBAs per unit mass were systematically calculated in the reaction system.

In the case of the same PBA concentration, the PBA mass efficiency rate is ranked in order from low to high: HCFC-141b, HFCO-1233zd(E), HFC-365mfc, HFO-1336mzzZ, and HFC-245fa. The order of PBA mass dissolution rate is just the opposite. The mass loss rate of the different PBAs did not show significant differences. Both the PBA mass efficiency and dissolution rate are controlled by the vaporization and condensation process of PBA. The ratio of the three states of each PBA relative to the total mass of material used, $r_{T}$ (wt%), shows a clear positive correlation with w. This indicates that the concentration of PBA is the dominant controlling factor for the absolute amount of each state of PBA.

For the same type of PBA, the amount of heat absorbed per unit mass of PBA decreases gradually as the PBA content increases. The relationship between both shows a pattern of rapid decrease followed by a slower decrease. The heat absorbed by PBA per unit mass is a key factor affecting the internal temperature of the foam when it stops expanding. Based on heat control of the polyurethane reaction system, the ranking of the five PBAs from best to worst was: HFC-245fa, HFC-365mfc, HFCO-1233zd(E), HFO-1336mzzZ, and HCFC-141b.

## Figures and Tables

**Table 1:** Parameters related to PBAs.

PBA	Molecular Weight	Critical Temperature/K	Critical Pressure/×10^5^ Pa	Eccentricity Factor
HCFC-141b	116.95	477.5	42.12	0.219
HFC-245fa	134.05	427.0	36.51	0.378
HFC-365mfc	148.07	460.0	32.66	0.377
HFCO-1233zd(E)	130.50	439.6	36.24	0.303
HFO-1336mzzZ	164.06	444.5	29.03	0.386

**Table 2:** Foaming recipes.

Ingredient	Weight/g				
R4110A	100	100	100	100	100
DMCHA	1.2	1.2	1.2	1.2	1.2
PC41	0.6	0.6	0.6	0.6	0.6
DBTDL	0.3	0.3	0.3	0.3	0.3
H2O	0.5	0.5	0.5	0.5	0.5
BL-8950	1	1	1	1	1
TCPP	20	20	20	20	20
PBA*	10	20	30	40	50
PM-200	208	208	208	208	208

**Table 3:** Fitted scale coefficients for PBAs.

PBA	A × 10^−3^	B	C × 10^−3^	D × 10^−5^	E × 10^−6^
HFC-245fa	1.420	−18.66	92.02	−20.01	16.25
HFC-365mfc	0.121	−1.553	8.277	−1.917	1.675
HFCO-1233zd(E)	0.445	−5.848	29.21	−6.428	5.298
HFO-1336mzzZ	0.326	−4.309	21.83	−4.832	4.000

**Table 4:** Boiling points and specific heat of vaporization of different PBAs.

PBA	HCFC-141b	HFC-245fa	HFC-365mfc	HFCO-1233zd(E)	HFO-1336mzzZ
Boiling Point/K	305.15	288.45	313.35	292.45	306.55
Specific heat of vaporization at Boiling Point/J·g^−1^	222.73	196.8	188.19	194.24	164.52
Specific heat of vaporization at 293.15 K/J·g^−1^	228.50	194.24	197.65	193.89	170.42

**Table 5:** $T_{F}$ (K) values for different PBAs.

w/wt%	$T_{F}$ (K)				
	HCFC-141b	HFC-245fa	HFC-365mfc	HFCO-1233zd(E)	HFO-1336mzzZ
3.02	400.35	394.36	396.11	396.99	398.17
6.03	392.21	383.99	388.42	389.20	389.41
9.05	386.52	374.58	378.06	380.22	382.83
12.06	378.69	365.33	371.55	372.76	374.74
15.08	374.88	358.11	364.34	366.96	368.12

**Table 6:** Ideal gas specific heat capacity at constant pressure of PBAs at different concentrations.

w/wt%	$C_{p}^{\prime }$ (J·g^−1^·K^−1^)				
	HCFC-141b	HFC-245fa	HFC-365mfc	HFCO-1233zd(E)	HFO-1336mzzZ
3.02	0.891	1.638	1.426	1.338	1.284
6.03	0.882	1.417	1.367	1.245	1.207
9.05	0.875	1.278	1.300	1.158	1.159
12.06	0.865	1.184	1.263	1.101	1.108
15.08	0.860	1.132	1.226	1.064	1.073

**Table 7:** Liquid constant pressure specific heat of PBAs at different concentrations.

w/wt%	$C_{p}$ (J·g^−1^·K^−1^)				
	HCFC-141b	HFC-245fa	HFC-365mfc	HFCO-1233zd(E)	HFO-1336mzzZ
3.02	1.359	2.626	2.041	2.097	1.950
6.03	1.336	2.241	1.946	1.928	1.808
9.05	1.320	2.008	1.840	1.779	1.722
12.06	1.301	1.850	1.784	1.684	1.636
15.08	1.292	1.761	1.729	1.622	1.577

## Data Availability

Data is contained within the article.

## Nomenclature

A	Corresponding proportion coefficient
B	Corresponding proportion coefficient
C	Corresponding proportion coefficient
D	Corresponding proportion coefficient
E	Corresponding proportion coefficient
$m_{c}$	Material total mass of the foaming start without PBA, g
$m_{d}$	The PBA dissolution mass, g
$m_{e}$	The PBA efficiency mass, g
$m_{l}$	The PBA mass loss, g
$m_{PBA}$	The PBA mass in the material total mass, g
$Q_{d}$	The heat absorbing by PBA dissolution mass, J
$Q_{e}$	The heat absorbing by PBA efficiency mass, J
$Q_{l}$	The heat absorbing by PBA mass loss, J
$Q_{m}$	The heat absorbed per unit mass of PBA, J·g^−1^
$Q_{PBA}$	The total heat absorbing by PBA during the foaming process, J
R	Gas constant, J·mol^−1^·K^−1^
$r_{PBA}$	The ratio of each PBA’s three states to the total mass of PBAs used, %
$r_{dPBA}$	The dissolution rate of PBA relative to the total moles of PBA used, %
$r_{dT}$	The dissolution rate of PBA relative to the total mass of materials used, wt%
$r_{ePBA}$	The efficiency rate of PBA relative to the total moles of PBA used, %
$r_{eT}$	The efficiency rate of PBA relative to the total mass of materials used, wt%
$r_{lPBA}$	The loss rate of PBA relative to the total moles of PBA used, %
$r_{lT}$	The loss rate of PBA relative to the total mass of materials used, wt%
$r_{T}$	The ratio of the three states of each PBA relative to the total amount of material used, wt%
$T_{0}$	The initial foaming temperature, K
$T_{1}$	The temperature of PBA at the surface escape, K
$T_{r}$	Contrast temperature
$T_{r1}$	Contrast temperature in the state of “1”
$T_{r2}$	Contrast temperature in the state of “2”
$T_{F}$	Temperature inside foam when it stops expanding, K
w	The initial mass content of PBA in PU materials, wt%
Z	Compressibility factor
ω	Eccentricity factor
γ	The latent heat of vaporization of PBA at the temperature $T_{0}$, J·g^−1^
$(\Delta h_{v}{)}_{1}$	Enthalpy changes in the state of “1”, J·g^−1^
$(\Delta h_{v}{)}_{2}$	Enthalpy changes in the state of “2”, J·g^−1^
$C_{g0}$	Specific heat capacity of PBA gas at the temperature of $T_{0}$, J·g^−1^·K^−1^
$C_{g1}$	Specific heat capacity of PBA gas at the temperature of $T_{1}$, J·g^−1^·K^−1^
$C_{gF}$	Specific heat capacity of PBA gas at the temperature of $T_{F}$, J·g^−1^·K^−1^
$C_{L0}$	Liquid specific heat capacity of PBA at the temperature of $T_{0}$, J·g^−1^·K^−1^
$C_{LF}$	Liquid specific heat capacity of PBA at the temperature of $T_{F}$, J·g^−1^·K^−1^
$C_{p}$	Specific heat capacity at constant pressure for PBA in the liquid state, J·g^−1^·K^−1^
$C_{p0}$	Liquid specific heat capacity of PBA at temperature $T_{0}$, J·g^−1^·K^−1^
$C_{p}^{\prime }$	Specific heat capacity at constant pressure for PBA in its ideal gas state, J·g^−1^·K^−1^
$C_{p0}^{\prime }$	Ideal gas specific heat capacity of PBA at temperature $T_{0}$, J·g^−1^·K^−1^
$C_{pF}$	Liquid specific heat capacity of PBA at temperature $T_{F}$, J·g^−1^·K^−1^
$C_{pF}^{\prime }$	Ideal gas specific heat capacity of PBA at temperature $T_{F}$, J·g^−1^·K^−1^
